# Prognostic significance of KRT19 in Lung Squamous Cancer

**DOI:** 10.7150/jca.51179

**Published:** 2021-01-01

**Authors:** Xun Yuan, Ming Yi, Bing Dong, Qian Chu, Kongming Wu

**Affiliations:** 1 Department of Oncology, Tongji Hospital of Tongji Medical College, Huazhong University of Science and Technology, 1095 Jiefang Avenue, Wuhan 430030, P.R. China.; 2 Department of Molecular Pathology, The Affiliated Cancer Hospital of Zhengzhou University & Henan Cancer Hospital, Zhengzhou 450008, China.

**Keywords:** KRT19, lung cancer, squamous cell carcinoma, prognosis

## Abstract

**Backgroud:** Keratin 19 (KRT19) is the intermediate filament that constitutes the cytoskeleton and regulates cell-cycle and cell death.

**Objective:** We aimed to assess whether KRT19 was involved in lung cancer development.

**Methods:** The expression of KRT19 in lung cancer was evaluated from mRNA expression on open databse and protein abundance on tumor tissue array.

**Results:** Using open microarray gene expression datasets and differential expression analysis, we found that KRT19 was upregulated in lung cancer compared with normal tissue. Further analysis suggested that KRT19 mRNA expression was correlated with tumor progression and overall survival in lung cancer patients. As KRT19 was overexpressed in adenocarcinoma (AC) and squamous cell carcinoma (SCC), we examined the prognostic value of KRT19 protein abundance by tissue microarray (TMA). The results suggested that protein expression of KRT19 was significantly associated with overall survival of SCC.

**Conclusions:** Giving the prognostic role of KRT19 in lung cancer, KRT19 could be considered as an potential molecular marker in lung cancer, especially in SCC.

## Introduction

Lung cancer, a heterogeneous group of malignancies, remains the leading cause of cancer-related deaths in the world. The different histological types of lung cancer are divided into two main groups, small cell lung cancer (SCLC) and non-small cell lung cancer (NSCLC), the latter includes adenocarcinoma (AC), squamous cell carcinoma (SCC) and large cell lung cancer (LCC). AC and SCC are the most common histological types of lung cancer and account for approximately 70% of lung cancers 1. Small cell lung cancer (SCLC) is usually reported to comprise about 15% of all lung cancer cases recorded 2. Improved understanding of the biology of lung cancer has resulted in the development of new biomarker-directed therapies targeting molecular alterations (eg, EGFR mutations, ALK rearrangements, ROS1 rearrangements, and BRAF V600E mutations), which have prolonged survival of lung cancer patients 3. However, the progress is slow, with about 5% improvements in 5-year survival rates for the past 20 years 4. Therefore, it is crucial to identify additional prognostic biomarkers to generate individualized treatment and follow-up schedules 5, 6.

There is an urgent need for a highly reproducible, inexpensive biomarker, which might help to identify patients at high risk for disease-progression, recurrence, metastasis and death 7. Hence, we analyzed gene expression pattern in publicly available cohorts of lung cancer patients and identified a signature of genes which might be involved in lung cancer development and prognosis of patients. Among the identified genes, KRT19 is a member of the keratin family, which is responsible for the structural integrity of epithelial cells, including bronchial epithelial cells 8, 9. KRT19 expresses in normal lung tissue, as well as lung cancer tissues 10. However, when necrosis occurs within the lung tumor, KRT19 degrades and fragments are released into the blood. This leads to an increase of the blood CYFRA 21-1 level 11, 12. Hence, the serum level of KRT19 fragments could be applied in the clinic as a tumor marker.

Many studies have investigated the prognostic value of serum tumor biomarkers, including CYFRA 21-1 in lung cancer 13-16. However, the value of KRT19 in lung cancer is still unknown. In the present study, we evaluated the significance of KRT19 in lung cancer from mRNA and protein level.

## Materials and methods

### Analysis of Gene Expression data

In order to determine whether genes were differentially expressed between primary lung tumor and normal lung tissue, differential gene expression analysis was performed by the SAM method using histological type as the variable 17. Gene lists were created using a cut-off of Q <0.05, >1.5-fold change. The raw data for differential expression analysis are available at ArrayExpress (http://www.ebi.ac.uk/arrayexpress/) with accession number E-GEOD-18842 and E-GEOD-27262.

Six other independent datasets, available through the Gene Expression Omnibus (GEO) database in public repositories and containing gene expression and clinical data, were analyzed to evaluate the expression and prognosis of the biomarkers (GSE31210, GSE32863, GSE19188, GSE3141, GSE14814, GSE30219) 18.

### Lung cancer tissue microarray and immunohistochemical staining

To evaluate the clinical significance of KRT19 in patients with lung cancer, commercially available tissue microarray (TMA) slide (LC811, Alenabio, Inc., Xian, China) with pathological characteristic information was purchased for immunohistochemistry (IHC) analysis 19. Other tissue microarrays (HLug-Ade150Sur-02 and HLug-Squ150Sur-01, Shanghai Biochip Co., Ltd., Shanghai, China) both with 75 matched pairs of primary lung cancer samples and adjacent lung tissues were applied to evaluate the prognostic value of KRT19 based on its detailed survival data. Specific primary antibody against KRT19 (polyclonal rabbit antibody, 1:100; ProteinTech Group, Chicago, USA) was used for IHC with a 2-step protocol. Immunohistochemical staining was performed using a standard technique, as previously described 20, 21. Level of KRT19 expression was measured using Image-Pro Plus® software version 6.0.0.260.

### Statistical analysis

The Student's t-test and one-way ANOVA were applied to evaluate the differences in groups as appropriate and the significance level was set at 0.05. For the univariate analysis, patients' clinical end points were calculated using the Kaplan-Meier method and compared by the log-rank test. Forward stepwise multivariate Cox proportion hazard analysis was performed to determine the influence of age, gender, histologic subtype and TNM stage on overall survival (OS). Hazard ratios (HRs) with corresponding 95% confidence intervals (CIs) were estimated from the Cox analysis. We also employed Harrell's concordance index (C-index) to assess the model's prognostic accuracy in the multivariate analysis. A two-sided P<0.05 was considered statistically significant. All statistical analyses were performed with R, version 3.6.2 (http://www.r-project.org/).

## Results

### Transcriptional levels of KRT19 characterize lung cancer

In an effort to characterize histological type-specific gene signature of lung cancer, we analyzed gene expression patterns in microarrays through a method, Significance Analysis of Microarrays (SAM). Using a public dataset GSE18842 with 50 primary lung tumor and 50 paired adjacent lung tissues, SAM identified differential expression genes that changed at least 1.5-fold. Among them, KRT19 were significantly overexpressed in lung cancer (Table 1). It was also validated in another dataset GSE27262 with 25 primary lung tumor and 25 paired adjacent lung tissue.

**Figure 1:**
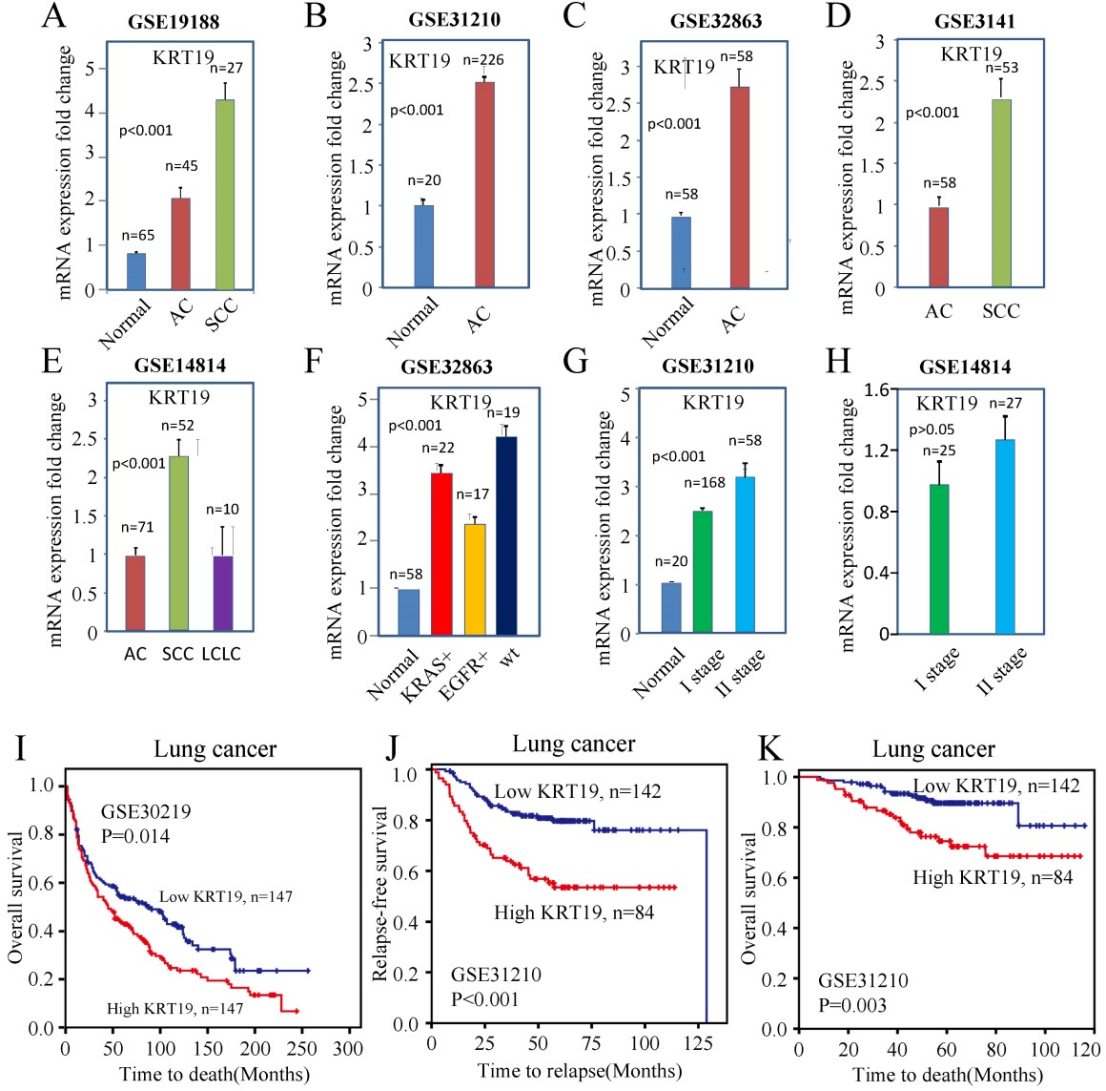
** The mRNA expression of KRT19 in lung cancer and normal tissue.** (A-C) KRT19 mRNA were overexpressed in tumor compared with normal lung tissue. (D-E) KRT19 mRNA was overexpressed in SCC. (F) KRT19 mRNA level was higher in wt lung cancer than in EGFR mutated lung cancer. (G-H) KRT19 expression increased with the presence of higher stage. (I) KRT19 expression was associated with OS in lung cancer patients from GSE30219. (J-K) KRT19 expression was associated with RFS and OS in lung cancer patients from GSE31210.

Then, we evaluated KRT19 transcript levels in microarrays downloaded from ArrayExpress dataset. In accordance with previous conclusion, the relative abundance of KRT19 mRNA increased 2- to 3-fold when normal tissue transformed into tumor (*t*-test, *p*<0.001) (Figure 1A-C).

To investigate the association between the KRT19 with distinct lung cancer subtypes, we compared KRT19 expression with subtypes of lung cancer in microarrays. The expression of KRT19 was higher in SCC than in AC and LCLC (Figure 1D-E). In addition, KRT19 mRNA was increased about 2-fold in *wild type* (wt) lung cancer compared with EGFR mutated lung cancer, suggesting that EGFR pathway might affect KRT19 gene expression (Figure 1F).

In order to test whether KRT19 is associated with lung cancer progression, we examined the association of KRT19 expression with TNM staging. An increased expression of KRT19 was correlated with higher TNM stage (Figure 1G-H). These analyses indicated that KRT19 could be a tumor marker for diagnosis of lung cancer, especially of SCC.

To understand the value of KRT19 in the prediction of lung cancer's prognosis, we examined expression of KRT19 in microarrays which provided survival data. The results indicated that KRT19 was statistically associated with the relapse-free survival (RFS) and OS rate of lung cancer (Figure 1I-K). Combined, these results revealed that KRT19 could be a diagnostic and prognostic biomarker of lung cancer.

### Tissue expression of KRT19 protein characterize lung cancer

**Figure 2:**
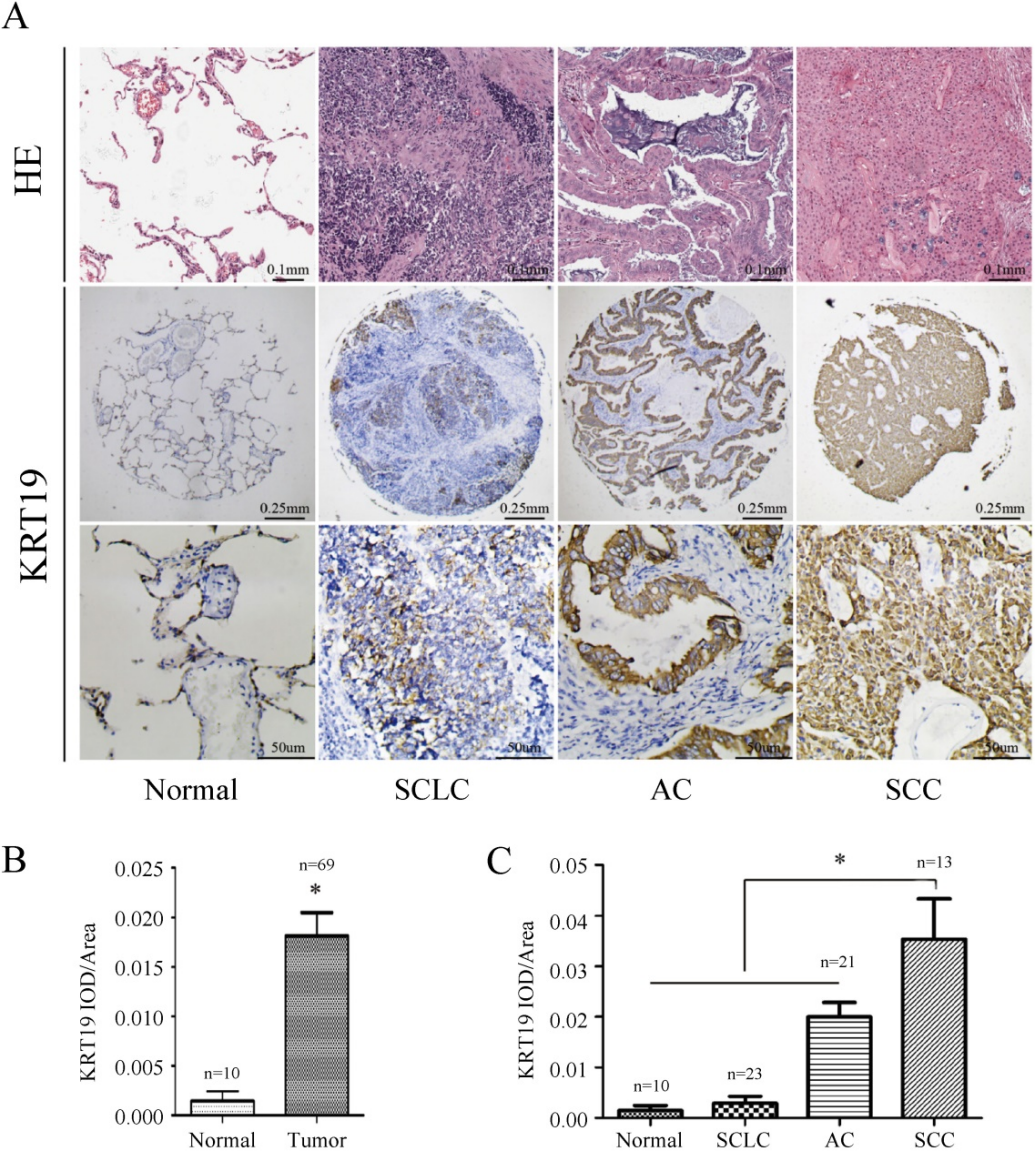
** Immunohistochemistry analysis of KRT19 in lung cancer tissues.** Representative images of KRT19 expression in normal lung tissue and different historical type of lung cancer were shown (A) with quantitative result displayed as mean ± SE (B-C).

In order to further explore the role of KRT19 in lung cancer, we analyzed KRT19 protein levels in a TMA containing 69 informative patients with distinct histological subtypes and additional normal lung tissues. KRT19 protein was predominantly detected in the cytoplasm, and representative images of immunohistochemical staining for noncancerous and cancerous tissue are shown in Figure 2A. We examined the potential association of KRT19 expression with the clinicopathological parameters by using quantitative criteria (Table 2). Statistical significance was found for KRT19 expression in AC, SCC and SCLC (*f-*test, *p*<0.001). Importantly, KRT19 was significantly overexpressed in SCC compared with AC (*t-*test, *p*=0.039). Immunohistochemical staining confirmed that KRT19 expression was high in lung tumor, especially in SCC (Figure 2B-C).

**Figure 3:**
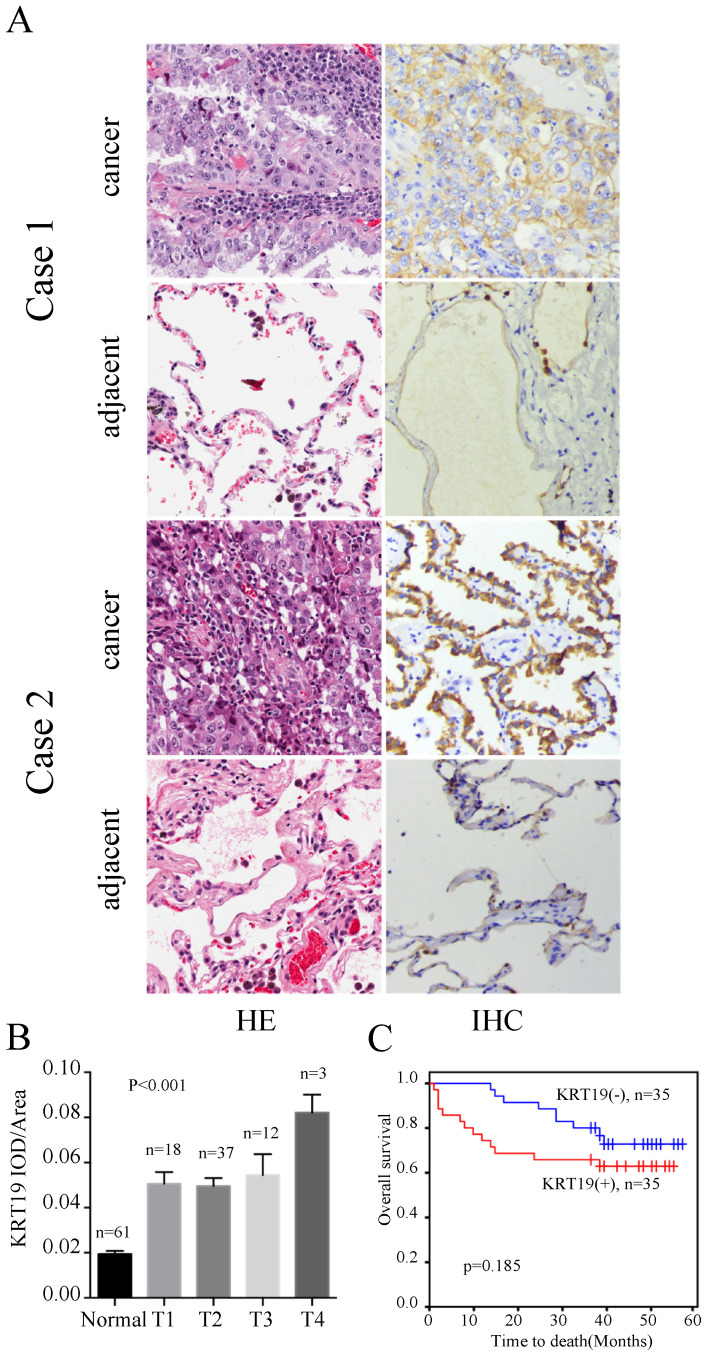
**Immunohistochemistry analysis of KRT19 in AC patients.** In a five years cohort, representative images of the cancerous and adjacent tissue from the longest survival patient and the shortest one were shown by using HE and IHC staining (A). The quantification of KRT19 expression in different stages was displayed as mean ± SE (B). Kaplan-Meier survival curve of KRT19 (C) was also analyzed.

To understand the value of KRT19 in the prediction of ACs' prognosis, we examined expression of KRT19 in the other tissue microarray, which provided survival data. The profiles of KRT19 of two representative cases were shown in Figure 3A. Case1 had the longest survival and case2 had shortest survival in this cohort. Intriguinly, expression of KRT19 was strongly correlated with the tumor T stage (Figure 3B). However, segregation of the patients into KRT19-high and low expression groups did not reveal significant relationship with clinicopathological parameters of sex, age, tumor size, location, grade (Table 3). We also investigated the association between cumulative overall survival-rates and clinicopathological factors by univariate and multivariate Cox regression analysis. As shown in Table 4, tumor size was prognostic factor for OS, whereas other clinicopathological factors were not directly related to the clinical outcome of AC. Besides, KRT19 expression was not correlated with OS in AC (Figure 3C).

**Figure 4:**
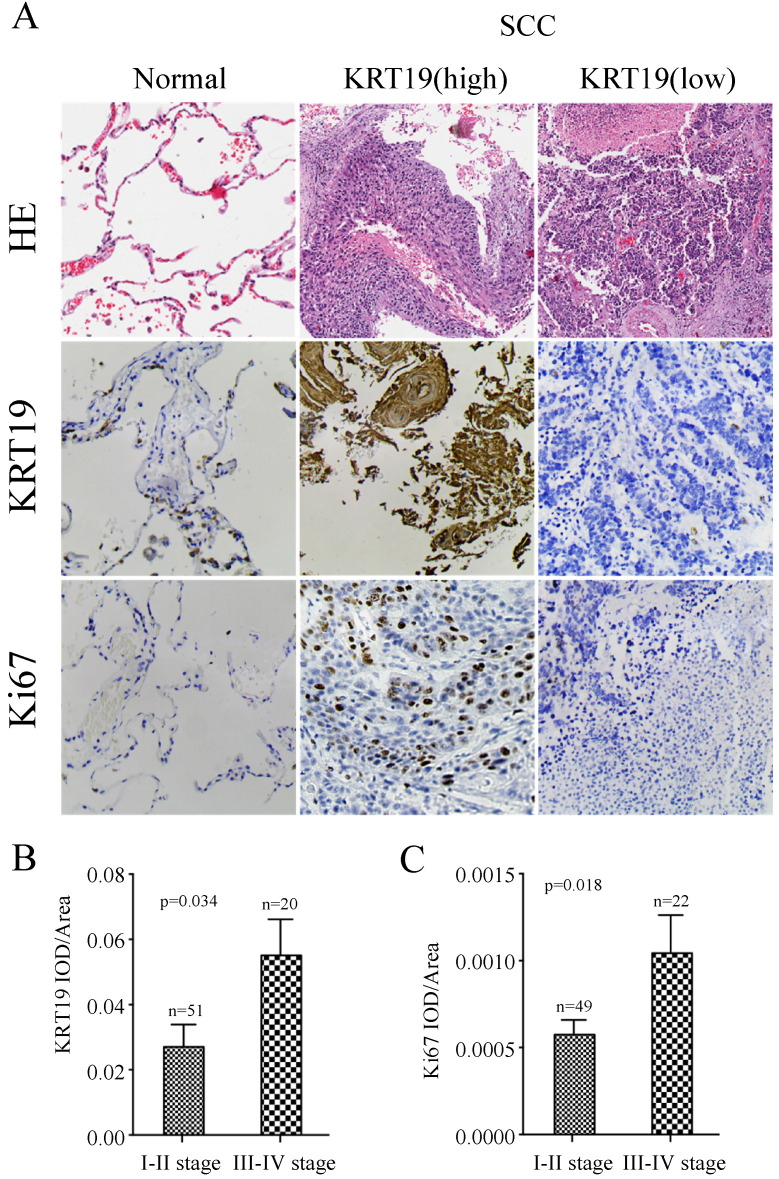
** Immunohistochemistry analysis of KRT19 in SCC tissues.** In a five years cohort, representative images of KRT19 expression in normal lung tissue and SCC were shown (A) with quantitative result displayed as mean ± SE (B-C).

**Figure 5:**
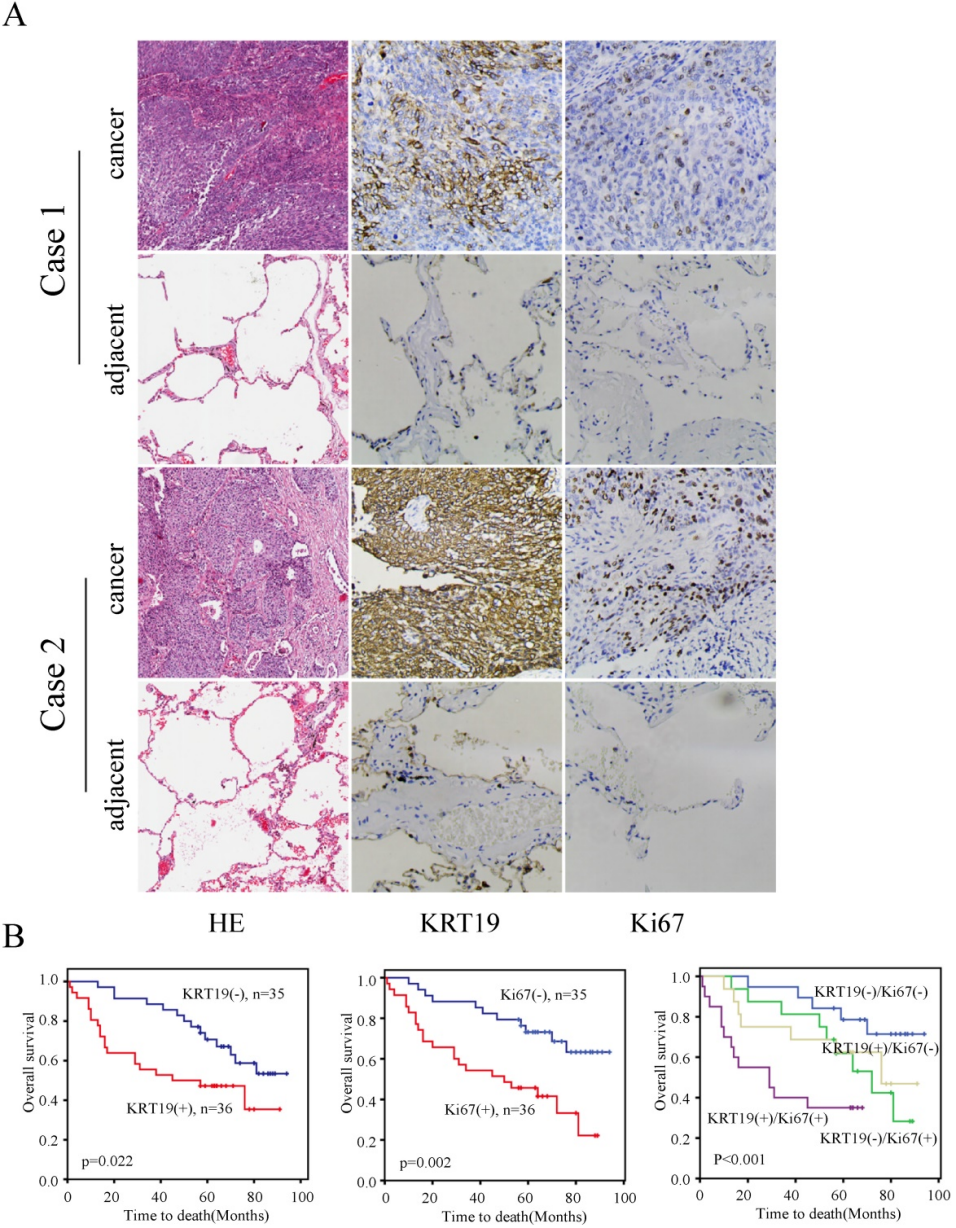
** KRT19 is an independent prognostic factor of overall survival in SCC patients.** In a five years cohort, representative images of the cancerous and adjacent tissue from the longest survival patient and the shortest one were shown by using HE and IHC staining (A). Kaplan-Meier survival curve of KRT19 alone or in combination with Ki-67was also analyzed (B).

To further explore the prognostic role of KRT19 in SCC, we employed another tissue microarray containing 70 SCC patients with complete survival data and detailed information about Ki-67 expression. The profiles of KRT19 and Ki-67 of three representative cases were shown in Figure 4A, which showed the positive correlation between KRT19 and Ki-67. In comparison of early-stage lung cancer tissue, the majorty of late-stage lung cancer samples showed increased expression of KRT19 and Ki-67 in this five years cohort (Figure 4B-C). Segregation of these patients into KRT19-high and low expression groups did not reveal significant relationship with clinicopathological parameters of sex, age, tumor size, location, but noticeably associated with tumor grading, TNM staging (Table 5). Univariate and multivariate Cox regression analysis were perfomed to analyze the association between overall survival-rates and clinicopathological factors. Age, TNM stage, Ki-67 and KRT19 were prognostic factors for OS, whereas other clinicopathological factors were not directly related to the clinical outcomes of SCC (Table 6). Two representative cases of the longest survival patient and the shortest survival patient were shown in Figure 5A. As expected, high expression of KRT19 and Ki-67 tended to predict worse clinical outcome (Kaplan Meier log-rank test, *p*=0.022; *p*=0.002). Combined examination of KRT19 with Ki-67 would provide more precise information for the clinical outcome of SCC patients. We performed the forward variable-selection procedure using the two factors and patients characterized by low levels of KRT19 and Ki-67 had the longest OS in this study (Kaplan Meier log-rank test, *p*<0.001).

## Discussion

KRTs are intermediate filament proteins that constitute the cytoskeleton and regulate cell-cycle, apoptosis, cellular response to stress and death through dynamic interactions with a range of cellular proteins, including kinases, receptors, adaptors, and effectors 22. There are 20 types of KRTs, as classified by the molecular weight and isoelectric point. These KRTs are categorized to 2 subtypes: low-molecular weight acidic type I and high-molecular weight basic or neutral type II 23. KRT19 is the smallest (40 kDa) known type I KRTs and has been related to some diseases 24. In a mouse model, knockdown of KRT19 caused skeletal myopathy via mitochondrial and sarcolemmal reorganization 25. Subsequently, KRT19 was identified as tumor-associated biomarker in various cancers, including esophageal squamous cell carcinoma, cervical cancer, breast cancer and HCC 26-29. Gene amplification of KRT19 was found in HER2-positive breast tumor, indicating a relationship between KRT19 and HER2 30. Then, it was found that KRT19 could interact with HER2 on the cell membrane and stabilize HER2. However, inhibition of KRT19 downregulated HER2 by increased ubiquitination and destabilization of HER2, and reduced cell viability 31. MicroRNA-492, originated from KRT19, showed significant association with the liver enzyme BAAT and was overexpressed in metastatic hepatoblastoma (HB) 32. In addition, it was found that KRT19 correlated with tumor size, tumor differentiation, metastasis and micovascular invasion of HCC. Mechanically, KRT19 increased invasiveness and induced chemoresistance in HCC cells 33. Furthermore, expression of KRT19 was associated with survival of HCC patients 34.

Importantly, KRT19 can be used as a molecular marker for reverse transcriptase PCR (RT-PCR) and ELISA-mediated detection of disseminated tumor cells in regional lymph nodes (LNs), peripheral blood (PB) and bone marrow from susceptible cancerous patients 12, 35-36. Recently, KRT19 was also used as a novel marker by circulated tumor cells (CTC) detection to identify tumor cells in breast cancer and colorectal cancer 37-39, and its positivity was demonstrated to be a prognostic indicator 40. However, KRT19 has not been well characterized in lung cancer, specifically with regard to the prognostic value. Our study has found that KRT19 is associated with clinical progression and is predictive of clinical outcomes in lung cancer as a whole in gene transcription and protein expression level.

Using microarray gene expression datasets and differential expression analysis, KRT19 was found to overexpress in lung cancer compared with normal tissue. Moreover, KRT19 mRNA was significantly associated with the presence of SCC, EGFR *wt* lung cancer, higher stage and shorter survival time of lung cancer patients. It was reported that serum level of KRT19 was associated with the efficacy of EGFR-TKI treatment in NSCLC harboring EGFR mutation, and that patients with high KRT19 responded poorly to EGFR-TKI, leading to a shorter survival time 41. As KRT19 represents the extent of the EGFR *wt* component in lung tumor, EGFR-TKI's efficacy in NSCLC is poor in high serum KRT19 group compared with normal serum KRT19 group. Our study confirmed the difference of KRT19 expression in different molecular phenotype of lung cancer. As KRT19 correlated with EGFR *wt* lung cancer, KRT19 may be a valuable predictive marker for EGFR-TKI use. Further clinical trials are needed to validate the significance of KRT19 in EGFR mutated patients.

We also employed tissue microarray to validate the value of KRT19 in protein level. Distinguishing SCC from AC could provide better treatment decisions. However, the histological classification mainly depended on biopsy. In addition, biopsy occasionally leads to controversial diagnosis in lung cancer for the inherent histologic heterogeneity that exists in a subset of lung cancer 42. Our data indicated that KRT19 levels were much higher in SCC than in AC, and KRT19 showed the strongest correlation with stage and grade in SCC. Liquid bead array hybridization assay has been applied as a predictive and prognostic index of cancer patients and guiding clinical treatment, making KRT19 based CTC a promising diagnostic method for SCC. We speculated that interaction between KRT19 and other proteins including cell proliferatin marker Ki-67, initiated signaling cascades that promoted tumorigenesis, tumor invasion and metastasis. Rencently, KRT19 was found to interact with β-catenin/RAC1 complex and regulated NUMB and Notch1 expression, leading to the enhancement of the cancer stem-like cell properties in breast cancer 43. Furthemore, KRT19 expression was identified as an independent prognostic factor for the overall survival for SCC patients. Hence, the function of KRT19 in SCC is remarkable, and it would be interesting to explore the underlying mechanisms in detail.

In summary, KRT19 is a prognostic marker in lung cancer. High KRT19 expression in lung cancer was associated with clinical progression and could be used as a clinically relevant marker in lung cancer patients, especially in SCC patients. Recently, liquid biopsy has been used to identify cancer patients based on KRT19 expression. Further studies are needed to compare the predictive value of CTC detection and tumor tissue IHC in SCC.

## Figures and Tables

**Table 1:** Differential expression analysis identified genes KRT19 from GSE18842 and GSE27262

Gene Symbol	Gene Description	Dataset	Normal	Tumor	Fold Change	*p*-value
KRT19	Keratin 19	GSE18842	10.264	12.054	3.458	0.008
		GSE27262	10.663	11.648	1.979	0.003

**Table 2:** Association between biomarker expression and clinicopathological characteristics in TMA (LC811)

Characteristics	Total	High expression	
		KRT19	*P*-value
Number n (%)	69	35 (50.7)	-
**Age (years) (n [%])**			0.022
< 53	29 (42.0)	10 (34.5)	
≥ 53	40 (58.0)	25(62.5)	
**Gender (n [%])**			0.155
Female	19 (27.5)	7 (36.8)	
Male	50 (72.5)	28 (56.0)	
**Histology (n [%])**			<0.001
AC	33 (47.8)	21 (63.6)	
SCC	13 (18.8)	12 (92.3)	
SCLC	23 (33.4)	2 (8.7)	

**Table 3:** Association between biomarker expression and clinicopathological characteristics in AC TMA (HLug-Ade150Sur-02)

Characteristics	Total	High expression	
		KRT19	*P*-value
Number n (%)	70	37 (52.9)	
**Age (years) (n [%])**			0.808
< 60	29 (41.4)	14 (48.3)	
≥ 60	41 (58.6)	21 (51.2)	
**Gender (n [%])**			1.000
Female	34	17 (50.0)	
Male	36	18 (50.0)	
**Maximal tumor size (n [%])**			0.051
≤ 5 cm	53	23 (43.4)	
> 5 cm	17	12 (70.6)	
**Tumor location (n [%])**			0.626
Left	28	15 (53.6)	
Right	42	20 (47.6)	
**Grade (n [%])**			1.000
1+2	56	28 (50.0)	
3+4	14	7 (50.0)	

**Table 4:** Univariate and multivariate Cox regression analyses of multiple variables for OS (HLug-Ade150Sur-02)

Characteristics	OS			
	Univariate analysis		Multivariate analysis	
	HR (95% CI)	*P*-value	HR (95% CI)	*P*-value
**Age (years) (n [%])**				
< 60	1 (Reference)		1 (Reference)	
≥ 60	1.233 (0.517,2.940)	0.637	0.701 (0.269,1.824)	0.466
**Gender (n [%])**				
Female	1 (Reference)		1 (Reference)	
Male	1.903 (0.798,4.540)	0.147	1.434 (0.556,3.700)	0.456
**Maximal tumor size (n [%])**				
≤ 5 cm	1 (Reference)		1 (Reference)	
> 5 cm	3.686 (1.584,8.575)	0.002	5.147 (1.834,14.443)	0.002
**Tumor location (n [%])**				
Left	1 (Reference)		1 (Reference)	
Right	1.337 (0.540,3.313)	0.531	0.949 (0.365,2.468)	0.915
**Grade (n [%])**				
1+2	1 (Reference)		1 (Reference)	
3+4	0.971 (0.329,2.869)	0.957	0.359 (0.095,1.352)	0.130
**KRT19**				
Negative	1 (Reference)		1 (Reference)	
Positive	1.760 (0.751,4.123)	0.185	1.184 (0.477,2.943)	0.716

**Table 5:** Association between biomarker expression and clinicopathological characteristics in SCC TMA (HLug-Squ150Sur-01)

Characteristics	Total	High expression	
		KRT19	*P*-value
Number n (%)	71	36 (50.7)	
**Age (years) (n [%])**			0.727
< 64	33 (46.5)	16 (48.5)	
≥ 64	38 (53.5)	20 (52.6)	
**Gender (n [%])**			0.290
Female	4 (5.6)	1 (0.3)	
Male	67 (94.4)	35 (52.2)	
**Maximal tumor size (n [%])**			0.188
≤ 7 cm	61 (85.9)	29 (47.5)	
> 7 cm	10 (14.1)	7 (0.7)	
**Tumor location (n [%])**			0.734
Left	29 (40.8)	14 (48.3)	
Right	42 (59.2)	22 (52.4)	
**Grade (n [%])**			0.007
1+2	58 (81.7)	25 (43.1)	
3+4	13 (18.3)	11 (84.6)	
**TNM (n [%])**			0.048
I+II	49 (69.0)	21 (42.9)	
III+IV	22 (31.0)	15 (68.2)	

**Table 6:** Univariate and multivariate Cox regression analyses of multiple variables for OS (HLug-Squ150Sur-01)

Characteristics	OS			
	Univariate analysis		Multivariate analysis	
	HR (95% CI)	*P*-value	HR (95% CI)	*P*-value
**Age (years) (n [%])**				
< 64	1 (Reference)		1 (Reference)	
≥ 64	3.017 (1.405,6.479)	0.005	2.753 (1.190,6.369)	0.018
**Gender (n [%])**				
Female	1 (Reference)		1 (Reference)	
Male	0.411 (0.144,1.176)	0.097	0.244 (0.071,0.840)	0.025
**Maximal tumor size (n [%])**				
≤ 7 cm	1 (Reference)		1 (Reference)	
> 7 cm	1.224 (0.472,3.172)	0.677	0.787 (0.285,2.176)	0.645
**Tumor location (n [%])**				
Left	1 (Reference)		1 (Reference)	
Right	0.751 (0.380,1.481)	0.408	0.578 (0.279,1.196)	0.139
**Grade (n [%])**				
1+2	1 (Reference)		1 (Reference)	
3+4	1.568 (0.679,3.619)	0.292	0.632 (0.237,1.682)	0.358
**TNM (n [%])**				
I+II	1 (Reference)		1 (Reference)	
III+IV	2.232 (1.106,4.504)	0.025	2.119 (0.920,4.882)	0.078
**Ki-67**				
Negative	1 (Reference)		1 (Reference)	
Positive	2.575 (1.263,5.248)	0.009	2.686 (1.191,6.059)	0.017
**KRT19**				
Negative	1 (Reference)		1 (Reference)	
Positive	2.206 (1.097,4.437)	0.027	2.904 (1.269,6.643)	0.012
